# A cost-effective approach to DNA methylation detection by Methyl Sensitive DArT sequencing

**DOI:** 10.1371/journal.pone.0233800

**Published:** 2020-06-04

**Authors:** Wendell Jacinto Pereira, Marília de Castro Rodrigues Pappas, Dario Grattapaglia, Georgios Joannis Pappas

**Affiliations:** 1 Department of Cell Biology, University of Brasília, Brasília, Distrito Federal, Brazil; 2 Embrapa Genetic Resources and Biotechnology, Brasília, Distrito Federal, Brazil; 3 Universidade Católica de Brasília, Brasília, Distrito Federal, Brazil; University of California San Diego, UNITED STATES

## Abstract

Several studies suggest the relation of DNA methylation to diseases in humans and important phenotypes in plants drawing attention to this epigenetic mark as an important source of variability. In the last decades, several methodologies were developed to assess the methylation state of a genome. However, there is still a lack of affordable and precise methods for genome wide analysis in large sample size studies. Methyl sensitive double digestion MS-DArT sequencing method emerges as a promising alternative for methylation profiling. We developed a computational pipeline for the identification of DNA methylation using MS-DArT-seq data and carried out a pilot study using the *Eucalyptus grandis* tree sequenced for the species reference genome. Using a statistic framework as in differential expression analysis, 72,515 genomic sites were investigated and 5,846 methylated sites identified, several tissue specific, distributed along the species 11 chromosomes. We highlight a bias towards identification of DNA methylation in genic regions and the identification of 2,783 genes and 842 transposons containing methylated sites. Comparison with WGBS, DNA sequencing after treatment with bisulfite, data demonstrated a precision rate higher than 95% for our approach. The availability of a reference genome is useful for determining the genomic context of methylated sites but not imperative, making this approach suitable for any species. Our approach provides a cost effective, broad and reliable examination of DNA methylation profile on *Msp*I/*Hpa*II restriction sites, is fully reproducible and the source code is available on GitHub (https://github.com/wendelljpereira/ms-dart-seq).

## 1. Introduction

In the context of a common DNA sequence, cells employ different strategies to coordinate the set of expressed and repressed genes in order to establish cell identity and fate during development and physiological state, as well as adaptation to external stimuli. Epigenetics is defined in this context, and for decades is galvanizing the attention of the scientific community as a tunable adaptive layer modulating phenotypes of a given genotype [1]. Notwithstanding, this view of epigenetics is still debatable because it does not necessarily include transgenerational inheritance [2].

Epigenetic information is intertwined with chromatin accessibility and several effectors are involved as DNA methylation, post-translational modifications of histone tails and selected non-coding RNAs. It is also observed that these effectors act synergistically to reinforce the overall chromatin packing density, which in turn influences the transcriptional state of genes [3,4].

The earliest and probably most recognizable of the epigenetic marks is DNA methylation, that is the result of the addition of a methyl group in the 5’ position of cytosines (5mC) by several conserved and lineage specific DNA methyltransferases [5]. In mammalian genomes these modifications occur mainly in the CG sequence context, but in other organisms, particularly plants, other variations are also observed, such as CHG and CHH (H = A, C or T). Although other forms of base modifications are recognizable and associated with important roles [6], cytosine methylation mark is the most abundant chemical tag in eukaryotic DNA [7]. While base identity remains the same, 5mC is actively perceived in the cellular milieu exerting profound influence in the genome, which prompted its designation as DNA’s fifth base [8]. Despite its overwhelming occurrence, it is important to highlight that methylation is not universal in eukaryotes, given that 5mC appears to be absent in model organisms such as *Saccharomyces cerevisiae*, *C*. *elegans* and *Drosophila melanogaster* [7].

Regions enriched in 5mC are generally associated with closed chromatin state and therefore transcriptionally repressed [9]. In constitutive chromatin, high levels of DNA methylation decorate the repetitive fraction of the genome, such as transposable elements (TE) and satellite DNA. This plays a fundamental role in chromosome stability and genome integrity, maintaining TEs in a silenced state [10]. In the vicinity of genic regions, the degree of methylation can have contrasting outcomes regarding gene expression. Transcription is blocked if the promoter region is methylated [9]. Conversely, 5mC incidence in exonic regions is a distinctive feature of a subset of genes that are moderately expressed and constitutive [5,11]. Known as gene body methylation (GBM), this phenomenon, albeit taxonomically widespread, is not necessary for viability and its precise role is not clearly defined [12], although it has been implicated in helping splicing and avoiding spurious transcription start sites [13].

Given the high prevalence and functional implications of 5mC, its detection is the most used avenue to investigate epigenetic phenomena. Methylation profiling can be used as a proxy to detect dynamic changes in chromatin structure associated with development, environmental clues or physiological/pathological states of cells and individuals. For this purpose, several ingenious experimental approaches, boosted by the advent of next-generation sequencing (NGS), were deployed over the years to interrogate cytosine methylation status with varying degrees of resolution [14–16]. In general, these methods can be classified in three classes: bisulfite conversion, affinity enrichment and restriction enzyme mediated filtration [17].

Treatment with sodium bisulfite deaminates unmethylated cytosine to uracil, whereas 5mC is not affected. Subsequent PCR amplification will replace the uracils by thymines. Reads from NGS libraries created in parallel with and without bisulfite treatment are mapped onto reference genomes and the contrasting C/T positions between the libraries indicate the degree of methylation at single base resolution. Whole-genome bisulfite sequencing (WGBS; also BS-seq or MethylC-seq) is considered the highest resolution method for 5mC profiling [8,18], yielding the most comprehensive DNA methylation map of a particular sample, i.e. the methylome. The extensiveness of WGBS paradoxically impairs its use due to high costs and requirement of a high-quality reference genome.

Alternatives to WGBS were developed to enable studies dealing with multiple samples, targeted methylation profiling and for species with poor genome resources. The solution is to narrow down the genome sampling by filtering out regions based on methylation density or sequence landmarks. Methylation profile techniques employing genome reduction, either based on restriction enzymes or affinity enrichment, sample a fraction of the genome and therefore decrease the sequencing effort and costs. As such, these methods represent cost-effective alternative approaches in comparison to WGBS, that requires high coverage (>30X) of whole genome sequencing and thus turn out to have prohibitive costs for large sample size studies [19]. Methods with general application fall into two broad categories: affinity filtering and restriction enzyme (RE) based. Affinity enrichment methods (reviewed in Zeng et al. [16]) rely on post DNA fragmentation capture of methylated regions in the genome using antibodies (MeDIP-seq; Down et al. [20]) or CpG binding proteins (MDB-seq, Li et al. [21]; MethylCap-seq, Brinkman et al. [22]), followed by NGS. The main drawbacks are the bias towards hypermethylated regions and the impossibility to ascertain the location of 5mC within the reads [17,23].

Genome reduction based on restriction enzyme sensitivity to methylated regions have been used for decades [24]. Using a frequent cutter enzyme such as *Hpa*II, that cleaves CCGG sites only if cytosines are not methylated, digestion fragments are size selected and subjected to NGS. Techniques such as methyl-sensitive cut counting (MSCC; Ball et al. [25]), Methyl-seq (Brunner et al. [26]) and HELP-seq (Oda et al. [27]) construct reduced representation libraries from *Hpa*II digestion using *Msp*I, its methylation insensitive isoschizomer, as a normalizing control. Even though these fragments represent a very small fraction of the genome, they are enriched in hypomethylated regions and relevant functional elements such as CpG islands, promoters and gene bodies [25,27]. To overcome the deficient sampling of CpGs imposed by *Hpa*II CCGG recognition site, other methyl sensitive restriction enzymes (MREs) were used to broaden the sampled loci, as is the case of the expanded MSCC [28] and MRE-seq [29]. Both methods reinforce the observation that, in mammals, promoters are hypomethylated in contrast with 5mC incidence in inter and intragenic regions. It was also shown a negative correlation between sites sampled by MRE-seq and MeDIP-seq, that both methods are accurate and can be used to appraise general methylation status, despite not being able to recognize individual 5mC [29].

Here, we applied a technique based on MRE-seq, named Methyl Sensitive DArT-seq (MS-DArT-seq), which is an adaptation of DArT sequencing (DArT-seq), a genotyping technology based on the combination of double digestion of genomes, followed by special adapter ligation and next generation sequencing [30–33]. This RE complexity reduction creates a reduced representation of the genome, by producing selective and reproducible fragments representing methylation loci, providing a cost-effective methylation profiling alternative to WGBS (revised in Xing et al. [19] and Paun et al. [34]). MS-DArT-seq implements methylation profiling by mirroring the procedure of MSCC, where two libraries are constructed in parallel using restriction enzymes that target CCGG sites and show contrasting methylation sensitivity (*Msp*I, methylation insensitive; *Hpa*II which does not cleave if the internal cytosine is 5’-methylated). Unlike MSCC, a double digestion with *Pst*I is employed in MS-DArT-seq and only doubly digested fragments (*Pst*I-*Msp*I/*Hpa*II) are selected by ligation of adaptors corresponding to the two RE overhangs. The enzyme *Pst*I also presents DNA methylation sensitivity, therefore, extending the sampling bias toward hypomethylated regions. As a proof of concept, we applied this technique to probe the DNA methylation status of thousands of sites in different tissues of a *Eucalyptus grandis* tree used to generate the reference genome of the species [35].

## 2. Results

### 2.1 MSD-tags and MSD-sites generation

Two libraries were constructed from each of three different tissues namely, juvenile leaves, adult leaves and developing xylem, using a pair of restriction enzymes for each (*Pst*I-*Msp*I and *Pst*I-*Hpa*II) and single-end sequenced using Illumina platform generating more than 45 million 77 base pairs (bp) reads. After processing and mapping the reads against the reference genome, 7,869,635 (17.40%) reads mapped in multiple positions and 5,282,139 (11.68%) did not align to the reference genome with the applied criteria. Thereby, 32,086,893 (70.92%) were unique mapped reads and used to derive the methylation status of the sequenced fragments.

**Fig 1:**
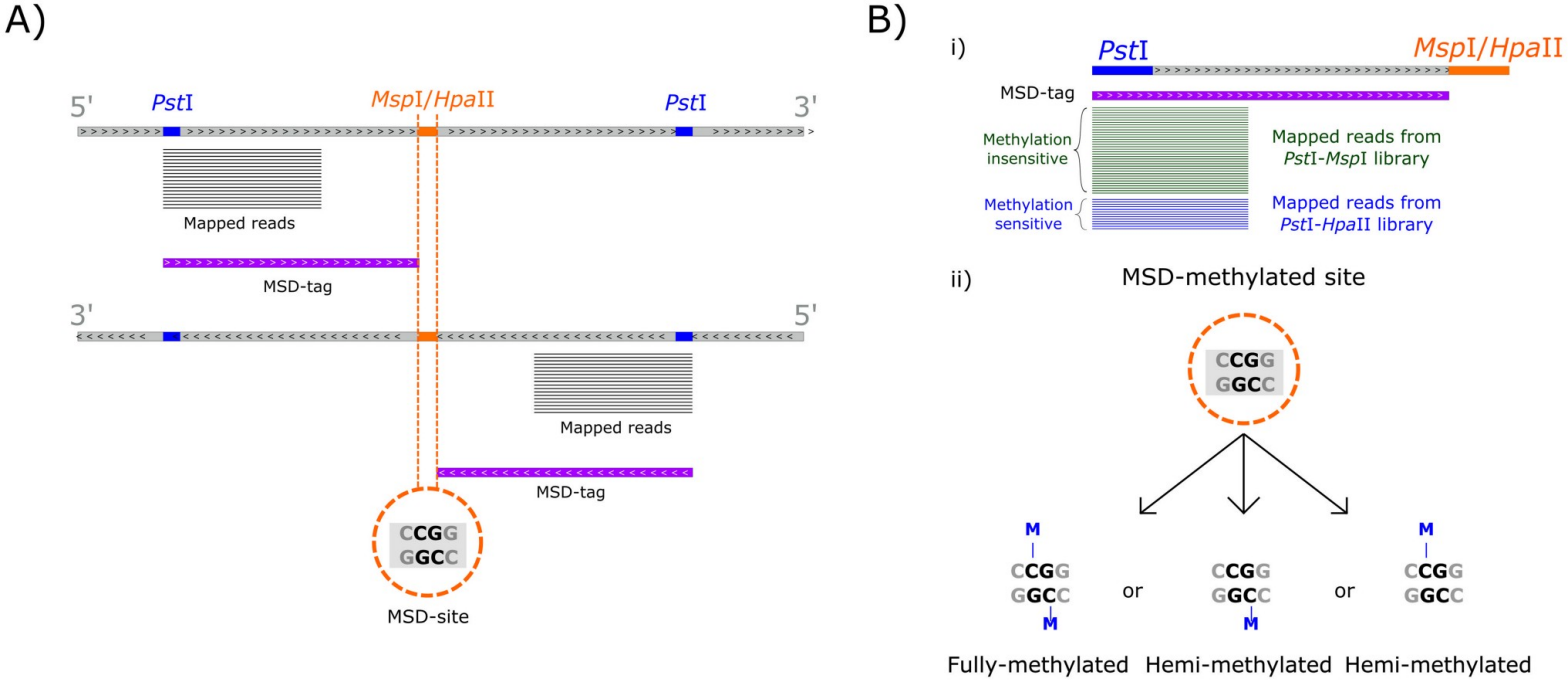
Illustration of MSD-tags and MSD-sites. A) A MSD-tag is defined as a sequenced DNA fragment whose count can be used to infer cytosine methylation status in the *Msp*I/*Hpa*II restriction site (named MSD-site). Notably, a MSD-site can be pictured by more than one MSD-tag, as portrayed in A). When MSD-tags counts for each library are significantly different (Fold change ≥ 2 and False Discovery Rate (FDR) ≤ 0.05 in both edgeR and DESeq2), they are considered MSD-methylated sites (B-i). Contrasting *Msp*I and *Hpa*II activities only informs about the methylation status of internal cytosines within MSD-sites. Moreover, a MSD-methylated site can represent a fully-methylated or one of two hemi-methylated states, since it is not possible to distinguish them (B-ii).

We defined a MSD-tag as a region that starts at a *Pst*I restriction site in a read mapping location and extends to the next *Msp*I/*Hpa*II restriction site in the same strand (Fig 1A). It is worth remembering that the entire region between the two sites, i.e., the MSD-tag, could be deduced considering the *Eucalyptus grandis* reference genome. Once these MSD-tags were defined, mapped reads were counted and served as a proxy to assess the methylation status of cytosines in the *Msp*I/*Hpa*II restriction site comparing read counts for each MSD-tag between *Pst*I-*Msp*I and *Pst*I-*Hpa*II libraries (Fig 1B). Lower read count in the *Hpa*II library is expected in methylated regions, due to the impaired digestion of 5mC. Conversely, no significant read count differences between libraries are expected in non-methylated regions. Once the counts of a MSD-tag differ between libraries, the MSD-site is classified as methylated (MSD-methylated site). However, it is important to notice that it is not possible to determine if a MSD-methylated site is fully-methylated or hemi-methylated (Fig 1B).

**Fig 2:**
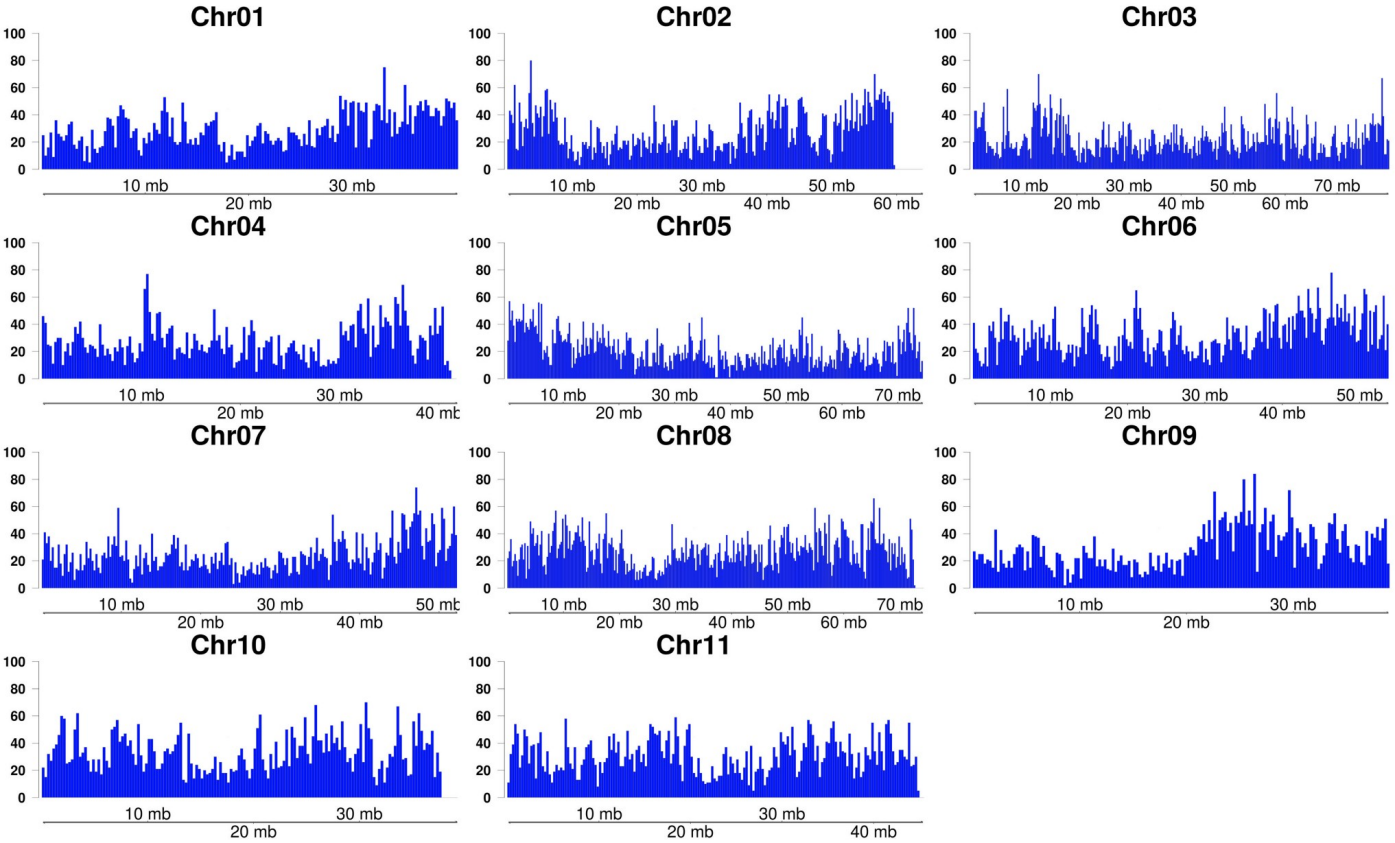
Distribution of 72,515 MSD-sites in the eleven *E*. *grandis* chromosomes. Each bar represents a window of 250 kb.

After processing MS-DArT-seq data, 76,106 unique MSD-tags were generated and had counts associated to each sample and library. Since multiple MSD-tags can represent the same restriction site, these 76,106 MSD-tags represents a set of 72,515 MSD-sites (internal cytosines in the *Msp*I/*Hpa*II restriction site). These MSD-sites were widely distributed in *E*. *grandis* genome covering all 11 chromosomes (Fig 2), albeit unevenly distributed. This was verified by a Pearson’s chi-squared goodness-of-fit test for the equality of the number of sampled sites along the chromosomes using various window sizes (see Methods). In all cases, the uniform distribution was rejected (p-value < 0.001).

### 2.2 Sampling breadth of MSD-tags

**Fig 3:**
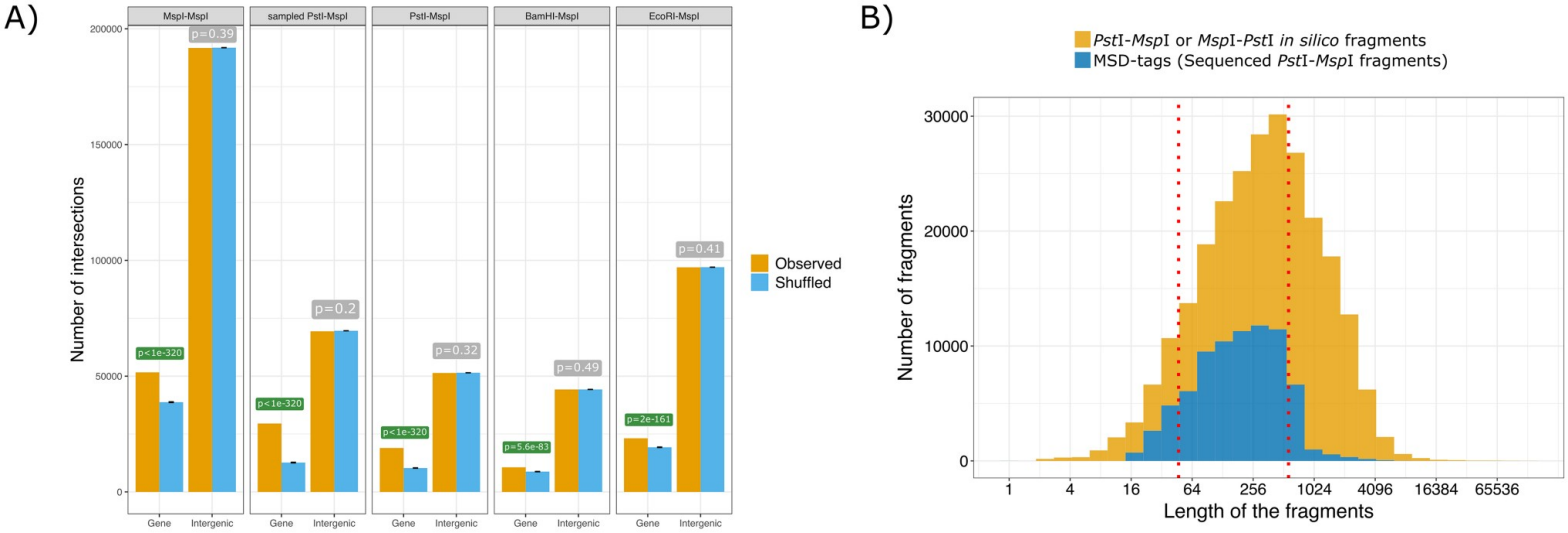
Distribution and co-localization of fragments generated by *in silico* digestion. A) Statistics of colocalization of *in silico* fragments generated by digestion with different combinations of REs and genomic features (genic and intergenic regions), as evaluated by the software Ologram [36]. In yellow, it is shown the observed intersections of the set of fragment intervals; in blue, intersections of the shuffled regions. Error bars represent the standard deviation of the shuffled distribution. The p-values for each feature colocalization is shown above the category bars. B) Distribution of fragments by size. In yellow, the distribution of fragments predicted by *in silico* digestion of the *E*. *grandis* genome by the combination of *Msp*I/*Hpa*II and *Pst*I restriction enzymes. Only fragments that have one end originated from a *Pst*I site and the other end from either a *Msp*I or *Hpa*II site are represented. In blue, 76,106 MSD-tags with reads in at least one of the sequenced libraries. The red lines show the 1^st^ and 9^th^ decile of the sequenced fragment sizes.

In order to assess the extent of genome reduction resulting from the double restriction digestion, we performed an *in silico* digestion of the reference genome. MS-DArT-seq reads were compared to the *in silico* digestion fragments to ascertain the degree of representativeness. We found that most MSD-tags (80%) have sizes within the range of 48 to 574 bp (Fig 3B), with an average of 289 bp (s.d. ~ 711.3, CV ~ 2.46). These MSD-tags account for 47.13% of the potential fragments in the same size range resulting from *in silico* double digestion. Since *Eucalyptus grandis* is a species with a high level of heterozygosity, the presence of SNPs in the reference genome may have prevented the detection of some restriction sites in our *in silico* analysis. Therefore, fragments extremely short or large are presumably artefacts. Consequently, the range between the 1^st^ and 9^th^ decile may represent a more reliable representation of the sampling capacity of the MS-DArT-seq.

The restriction enzyme *Pst*I is sensitive to DNA methylation, and this feature is leveraged by MS-DArT-seq methodology to evade highly methylated genomic regions, essentially enriching hypomethylated loci [32]. To investigate the breadth of MS-DArT-seq genomic reduction protocol, a computational simulation was carried out to probe for the genome-wide distribution of fragments resulting from an in silico double digestion using *Pst*I and *Msp*I/*Hpa*II. The program Ologram [36] was applied to test the statistical significance of the resulting fragments being colocalized with the set of genes in the genome. As a basal test, we evaluated the distribution of CCGG sites in the genome, i.e. fragments generated by *Msp*I digestion.

The results shown in Fig 3A ascertain that both the sequenceable fragment sets derived from either the *Msp*I single or double digestion with *Pst*I, show significant colocalization with genic regions, more than expected by chance (p-value<10^−320^). Simulations with other potential six-cutter REs, namely *Bam*HI and *Eco*RI, also show, to a lesser extent, significant colocalization with genes and no association with intergenic regions (Fig 3A). These observations suggest a skewed distribution of CCGG sites in the neighborhood and inside genes, accounting for the observed experimental bias of MRE-seq methods, including MS-DArT-seq.

### 2.3 Determination of DNA methylation

The determination of cytosine methylation status within the restriction sites was carried out by MSD-tag read counts comparison between *Pst*I-*Hpa*II and *Pst*I-*Msp*I libraries. The methylation sensitive *Pst*I-*Hpa*II library would, in principle, not yield reads in case of a fully methylated site but, given the heterogeneous methylation state of different cell types, *Hpa*II derived MSD-tags may be also sampled.

We adopted a statistical framework akin to RNA-seq differential gene expression to test for deviations in read counts per restriction site for the two libraries. The rationale is that, for a methylated RE site, *Msp*I derived read counts should be higher than the corresponding *Hpa*II derived.

**Fig 4:**
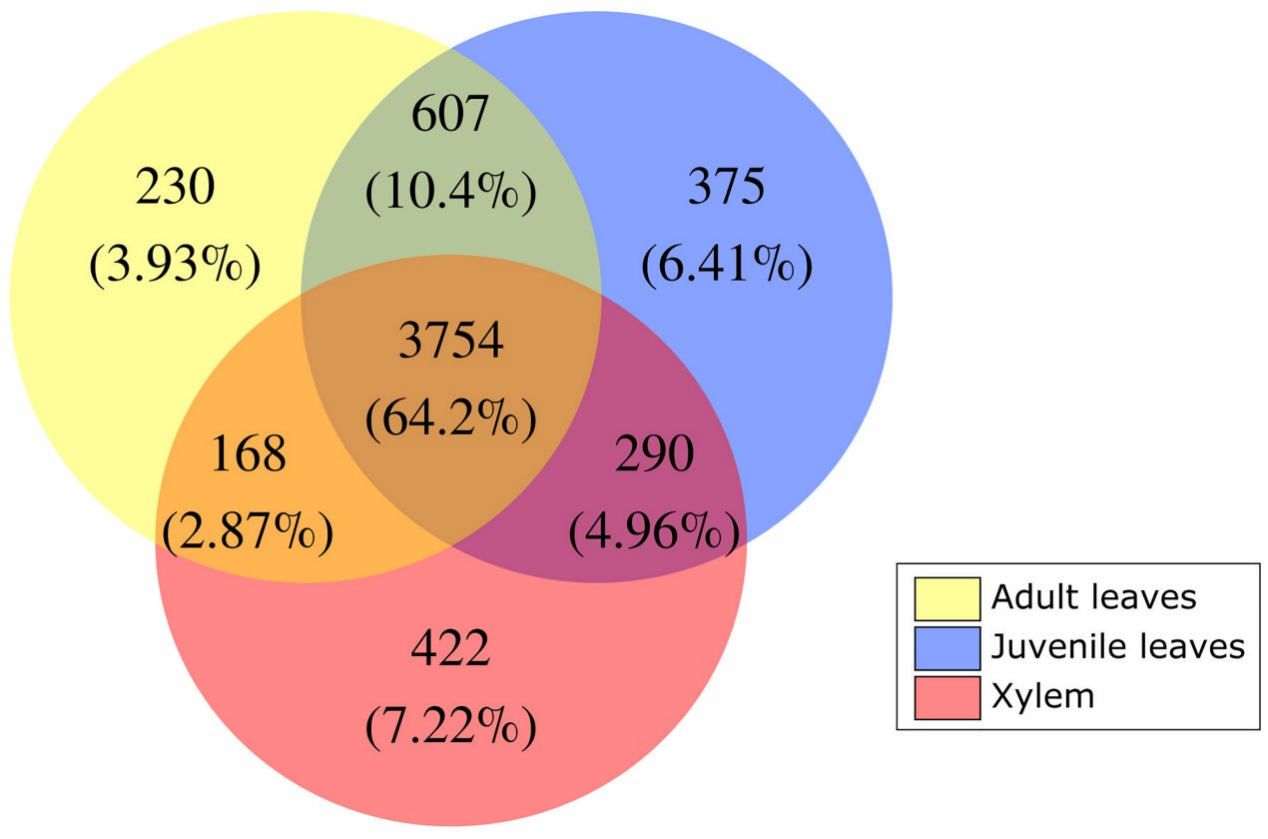
Venn diagrams demonstrating comparison among the MSD-methylated sites for all tissues.

First, we removed all MSD-sites with missing data for one or more of the tissues. Then, a minimum depth of 3 counts was imposed for a site to be considered. From a total of 76,106 MSD-tags generated for all BRASUZ1 samples, 32,357 (42.5%) were sampled in all tissues and 30,387 (39.9%) passed the criteria of minimal count. The read counts per MSD-tag were analyzed using both edgeR [37] and DESeq2 [38] to test for significant abundance differences between the two libraries. The MSD-tags for which both programs identified significant differences (fold change ≥ 2 and FDR < 0.05) were assumed to represent a truly MSD-methylated site. In total, 5,846 MSD-tags passed the criteria and, therefore, were considered as MSD-methylated sites (Fig 4).

### 2.4 Accuracy of DNA methylation detection using MS-DArT-seq data

We used whole genome bisulfite sequencing (WGBS) data of the adult leaves sample to certify the accuracy of MS-DArT-seq methylation calling. We considered only the 30,387 MSD-sites without missing data in any sample and with counts larger than 3 reads to keep the same criteria. A total of 4,759 MSD-methylated sites were identified in adult leaves sample using MS-DArT sequencing and 4,280 (89.94%) of these sites were sampled in WGBS. WGBS data shows 4,086 sites had at least one of the two internal cytosines classified as methylated (3,969 fully-methylated and 117 hemi-methylated). Considering only the MSD-methylated sites also covered by WGBS, the coincidence between the two approaches reached 95.47%, indicating a high efficiency of our analytical protocol in DNA methylation detection (S1 File).

One recurrent question about the use of the isoschizomers *Hpa*II and *Msp*I to detect DNA methylation is how to interpret profiles where fragments are more abundant in *Hpa*II library [39]. The pattern is normally attributed to hemi-methylation in one of the external cytosines of the restriction site. To investigate this issue, we applied the same analytical protocol to test if any MSD-site had counts significantly higher in *Pst*I-*Hpa*II than in *Pst*I-*Msp*I library. Only 47 MSD-sites showed this pattern in adult leaves sample. Twenty four (51%) of these sites are represented in WGBS, all of these completely unmethylated in WGBS data, except for one which had the internal cytosines in the fully-methylated state. Therefore, data produced in this study support the idea that sites whose fragments are more abundant in the *Hpa*II library appear to be spurious and do not represent hemi-methylation.

### 2.5 DNA Methylation distribution in BRASUZ1 tissues

MS-DArT-seq data were used to evaluate methylation patterns of selected tissues of BRASUZ1 tree: developing xylem, juvenile and adult leaves. Only the 32,357 MSD-sites without missing data in any tissue were used in this analysis. This subset of MSD-sites show similar distribution along the chromosomes as the full set (S1 Fig). Data reproducibility were assessed by comparison of MSD-tag counts in biological replicates for all the tissues sampled and each of the two libraries (*Hpa*II and *Msp*I). Within tissue correlation coefficients between replicates were high (*r* ≥ 0.97) and the same held between the two leaves samples of different developmental stages (S2 Fig). Principal component analysis using counts of MSD-methylated sites further highlights the methylation differences between xylem and leaves, even though not sufficient to differentiate young and adult leaves (S3 Fig).

Considering the three tissues sampled, a total of 5,846 MSD-methylated sites were detected. Separately, 4,759 MSD-methylated sites were detected in adult leaves sample, 5,026 in juvenile leaves and 4,634 in xylem (Table 1). Around 64.2% MSD-methylated sites are methylated in all tissues (Fig 4), reaching up to 74.6% of common MSD-methylated sites in adult and juvenile leaves. Despite this high intersection, sets of MSD-methylated sites exclusive of each tissue were also detected. Considering the set of 32,357 MSD-Sampled sites without missing data as a reference, it is possible to determine that the differences observed are larger than expected by chance (p-value < 0.001; Cochran’s Q test), with each tissue sample being significantly different from the others (p-value < 0.001 in all pairwise comparisons using Wilcoxon sign test).

**Table 1:** Genomic context of mapped MSD-methylated sites of BRASUZ1 tree by tissue sampled using MS-DArT sequencing.

Category	Subcategory	Adult leaves	Juvenile leaves	Developing Xylem	Total
**Genes**	**Intron or UTR**	803	826	817	923
	**Exon**	2,108	2,229	2,128	2,503
	**Total**	2,911	3,055	2,945	3,426
**TEs**	**Intron or UTR**	88	91	88	106
	**Intergenic**	689	737	565	886
	**Total**	777	828	653	992
**Feature overlap**	**Overlapping genes**	23	21	21	26
	**TE overlapping gene**	209	232	239	281
	**Total**	232	253	260	307
**Intergenic**	**Intergenic (outside TEs)**	839	890	776	1,121
**Total**		4,759	5,026	4,634	5,846

### 2.6 Genomic context of the MSD-tags

We set out to explore the genomic context of the MSD-methylated sites and, interestingly, observed that the majority were located in annotated genes (3,426 or 58.60%), from which 2,503 in exons and 923 in introns or UTRs. TEs accounted for 992 (16.97%) of the MSD-methylated sites location, 886 residing in intergenic regions and 106 representing TEs inside an intron or UTR of a gene. Around 19% (1,121) of MSD-methylated sites appear in regions without any annotation (Table 1). For 307 (5.20%) MSD-methylated sites, it was not possible to determine the genomic context, due to overlapping genes and/or TEs, reflecting possible annotation errors of *E*. *grandis* reference genome.

Other studies have demonstrated that genomic reduction by double digestion with enzymes *Pst*I-*Msp*I/*Hpa*II promotes a preferential sampling of gene rich regions [40,41]. Fisher’s exact tests were applied to determine if MS-DArT-seq sampling is biased toward genic space. Taking all possible *Msp*I/*Hpa*II restriction sites in *E*. *grandis* genome, it was found that MSD-sites are located within genes at a significantly higher proportion than expected by chance (p-value < 0.001; S1A Table). Moreover, considering only the set of MSD-sites without missing data sampled in this study by MS-DArT-seq (32,357), therefore excluding the influence of sampling bias, we determined that MSD-methylated sites occur more frequently within genes than in intergenic regions (p-value < 0.001; S1B Table).

**Fig 5:**
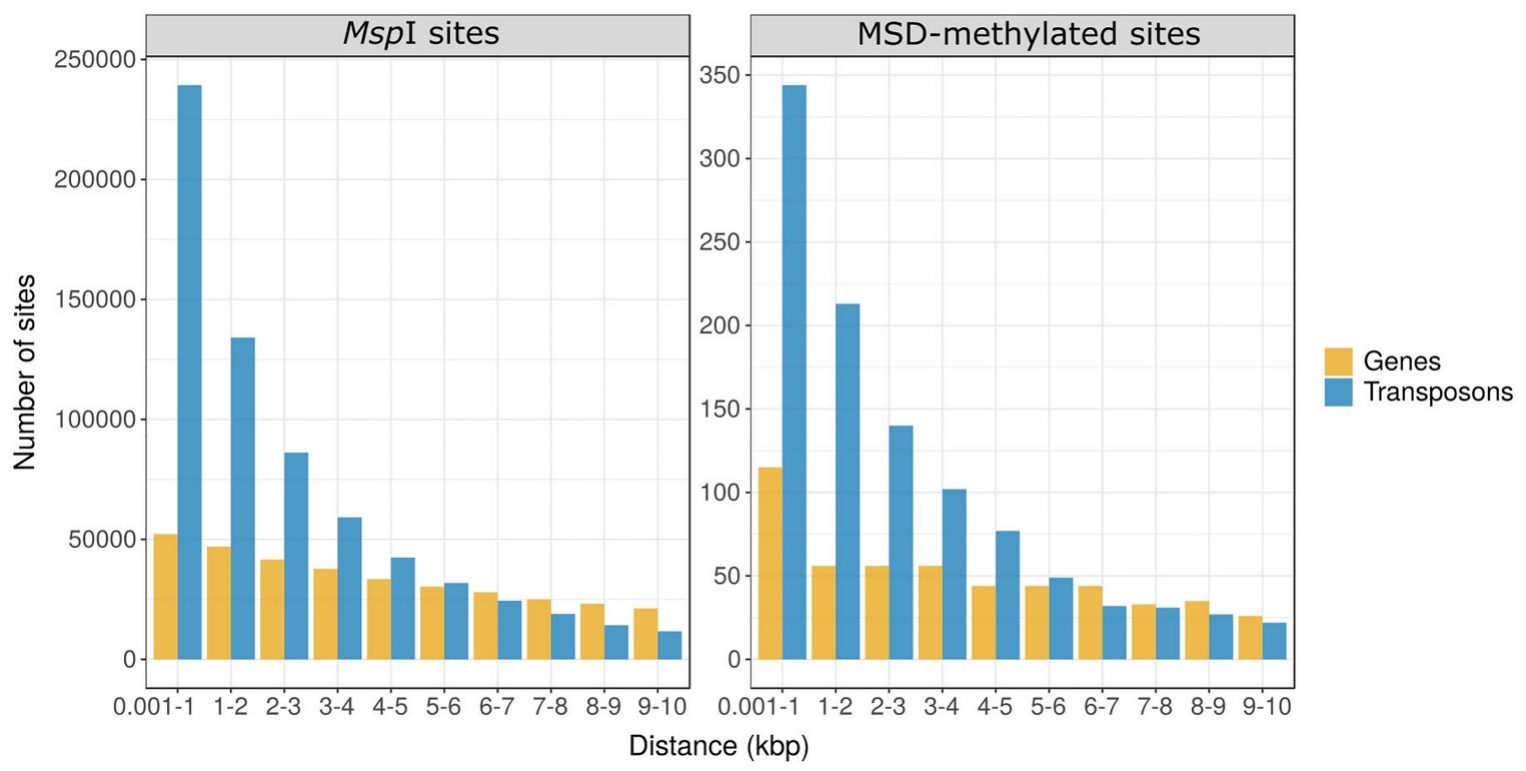
Distribution of *Msp*I/*Hpa*II sites present in the vicinity (10 kb) of genes and TEs (left) and distribution of MSD-methylated sites co-locating with genic regions (right).

Furthermore, the majority of the 1,121 MSD-methylated sites in intergenic regions are located within 10 kb from a gene (509 sites) or a TE (1,037 sites) as shown in Fig 5. In order to verify if there is a genic region enrichment bias promoted by MS-DArT-seq sampling, a chi-square test was applied to compare the distribution of these sites against the distribution of *Msp*I/*Hpa*II sites in the same interval of 10 kb of a gene or a TE (Fig 5, S4 Fig). Results demonstrated that concentration of MSD-methylated sites in the vicinity of genes is higher than expected only in the first kilobase, considering the proportion of *Msp*I/*Hpa*II sites at the same distance (p-value = 0.006; S4 Fig). Conversely, the incidence of MSD-methylated sites up to 10 kb from TEs is not biased in comparison with the distribution of *Msp*I/*Hpa*II sites in the same region (p-value = 0.52). Finally, comparison of MSD-methylated sites between tissues showed no significant difference regarding their occurrence near genes or TEs in the evaluated intervals.

**Fig 6:**
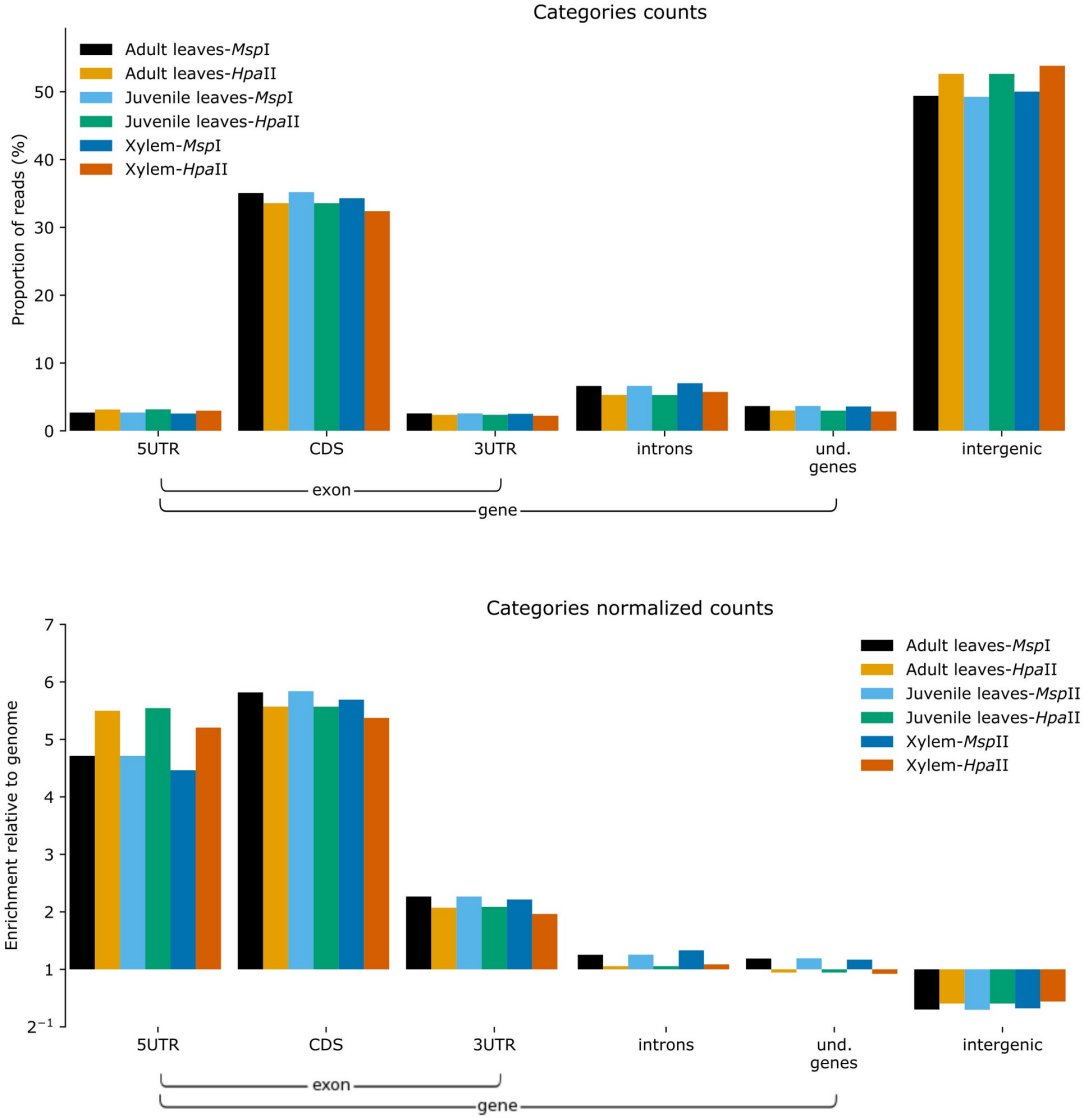
Distribution of MS-DArT-seq reads in the *E*. *grandis* genome accordingly with ALFA software. While the total number of reads are concentrated in intergenic regions (superior), the normalized counts reveal a clear bias of the MS-DArT-seq sampling towards genic regions (inferior). Because replicates counts from each tissue are similar, the sum is presented to simplify visualization.

Another evidence of the preferential sampling of gene rich regions were obtained applying the recently developed Annotation Landscape For Aligned reads (ALFA) software [42]. As shown in Fig 6, considering the normalized counts of the MS-DArT-seq mapped reads, there is a clear overrepresentation of genic regions, mostly 5’ UTR and coding sequences, while the intergenic regions are less represented than expected. Therefore, MS-DArT-seq promotes a cost-effective approach to the detection of DNA methylation in genic regions.

### 2.7 Comparison of tissue methylation profiles

Taking into account MSD-methylated sites present in genes (S5A Fig) or TEs (S5B Fig), it is possible to observe that the coincidence of MSD-methylated sites in genes among tissues is higher than what was found for all MSD-methylated sites (~ 75.4% and 64.2%, respectively). On the other hand, correspondence among MSD-methylated sites in TEs is reduced to approximately 48.2%. Once again, for both methylated genes and TEs (here defined as genes or TEs that contains at least one of the MSD-methylated sites), correspondence between leaves samples is higher than both general and the correspondence of any of the two distinct developmental stage leaves with xylem sample, reflecting the methylation patterns associated to tissue differentiation (S5C and S5D Fig).

Notwithstanding, it was also possible to detect MSD-methylated sites specific for each sample. To evaluate if distribution of MSD-methylated sites on specific genomic features is equivalent between the three samples, a chi-squared test was applied using the proportion of MSD-methylated sites in each category. The results show that there is a statistically significant association between tissues and MSD-methylated site genomic feature category (p-value ~ 0.04). The only significant distribution deviation demonstrates that xylem sample has less MSD-methylated sites in TEs located in intergenic regions than expected, as observed by Pearson standardized residuals (S6 Fig).

Since it is possible to have more than one MSD-methylated site per gene or TE, comparisons of methylated genes and TEs in different samples were also conducted (S5C and S5D Fig, respectively). Results of these comparisons were similar to MSD-methylated sites results with high correspondence of methylated genes among tissues (2,097 genes; 75.4%) and a lower correspondence of methylated TEs (406 TEs; 48.2%). However, the probability of a gene or TE be methylated depends on the tissue (p-value ~ 2.2^−06^ and p-value < 0.001, respectively; Cochran’s Q test). The Wilcoxon signed test applied to pairwise comparisons reveals that the set of methylated genes from juvenile leaves is different from both adult leaves and xylem sets (p-value < 0.001). However, the difference between the set of methylated genes from adult leaves and xylem are not significant (p-value ~ 0.92). Finally, it is possible to declare that the methylation patterns in TEs are different in all three evaluated tissues (p-value < 0.001 in all pairwise comparisons using Wilcoxon sign test).

#### 2.7.1 Classification and comparison of methylated TEs among tissues

**Fig 7:**
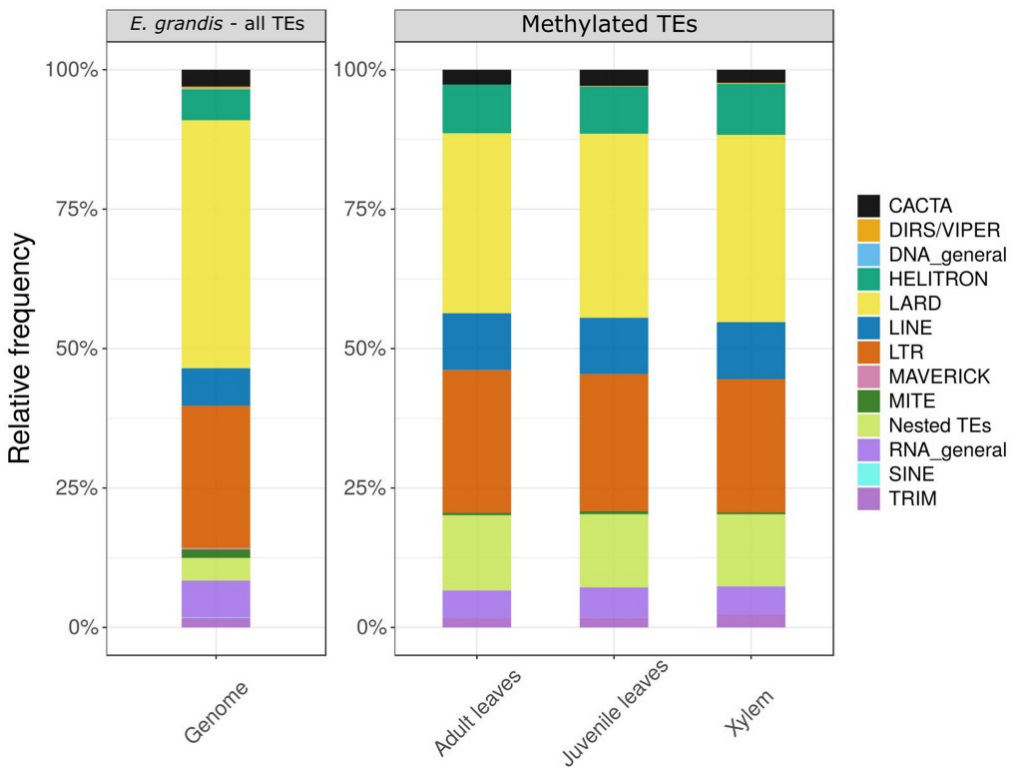
Relative frequency of all TEs in the *E*. *grandis* genome (left) and classes of methylated TEs in each sample (right). RNA_general and DNA_general are TEs not classified. Nested TEs reflects regions where more than one class of TEs that overlaps each other were predicted.

Transposable elements which had at least one MSD-methylated site were classified and their distribution were compared among samples (Fig 7; S7 Fig). To determine if the distribution of methylated TEs belonging to different classes is tissue dependent, and considering that the sets of methylated TEs differ among tissues, a chi-square test was applied using proportions of TEs in each class. Results showed that the proportion of methylated TE class does not differ among the samples (p-value = 0.99).

When the test was applied considering all annotated TEs as equally possible of being methylated, a significant difference was verified (p-value < 0.001), suggesting that some TE classes are not constitutively methylated as would be expected. By analysis of Pearson standardized residuals, it is possible to demonstrate that the three samples have fewer methylated TEs in LARD and MITE classes than expected (S8 Fig). Furthermore, all tissues sampled have more methylated LINE and Helitron TEs, as well as Nested TEs, than expected (Fig 7; S8 Fig). Thereby, maybe the methylation mechanism in the *E*. *grandis* species affect the different classes of TEs in distinct ways, since it was not possible to detect bias in the selection of sampled sites inside TEs in relation to TE’s class.

#### 2.7.2 Annotation and comparison of methylated genes among samples

All genes which have at least one MSD-methylated site, in one or more of the three BRASUZ1 samples investigated, were functionally annotated according to Gene Ontology (GO) (S2 File). To investigate if there is a class of genes preferentially methylated, an enrichment analysis of GO terms was performed. The results show that there are 149 enriched terms in the set of methylated genes verified in this study (S3 File), 74 (49.66%) present in all samples. Additionally, there are enriched terms in the set of methylated genes that are sample specific (S2 File). Most of these terms represents basic functions and processes. These results may reflect the fact that samples used in this study were trees growing in normal conditions without any stress or environmental challenge which could trigger a more specific set of methylated genes. Though, in juvenile leaves exclusive set, it is interesting to note that there are processes related to cell cycle, biosynthetic pathways and development in agreement with the sample developmental stage. Moreover, the results demonstrate the capacity of our approach to promote a complete functional annotation, that can be useful to deeply explore the biology behind a treatment or scenario where this approach can be applied.

## 3. Discussion

The featured MS-DArT-seq methodology is a 5mC profiling method that can be classified under the umbrella of genome reduction by means of methylation-sensitive restriction enzyme digestion, such as MSCC [25] or MRE-seq [29]. Our approach branches out from these established techniques as it relies on double digestion with the use of the RE *Pst*I and one of each of the isoschizomers *Msp*I (methylation insensitive) and *Hpa*II (methylation sensitive) that recognize the prototypical site (CCGG) covering CG and CHG methylation contexts. Two parallel libraries are constructed per sample using *Pst*I and one of each of the isoschizomers. Library preparation assures the directional selection of fragments containing both restriction sites (*Pst*I-*Msp*I/*Hpa*II) and the generation of a larger sequence context (up to 73 bases, mean of ~50 bases, after quality control) that improves genome mapping uniqueness when compared to MSCC’s short tags. The choice of *Pst*I is advantageous because it is also sensitive to methylation and its activity impaired in highly methylated areas, notably the repetitive fraction of genomes [32], thereby enriching hypomethylated regions such as transcriptionally active chromatin.

As a proof of concept, we used MS-DArT-seq to probe for tissue-specific methylation differences in the tree *Eucalyptus grandis*, although it can be applied to any species. Using three tissue samples, the straightforward protocol resulted in more than 30 thousand MSD-sites without missing data distributed along the 11 *Eucalyptus* chromosomes. It is important to mention that the DNA quality has an important role in the detection of MSD-sites, as poor-quality DNA will impair the full DNA digestion. In this study, DNA samples from xylem tissue had the smallest number of MSD-sites identified, a direct consequence of the poorer DNA quality of these samples compared to leaves. Therefore, we only used MSD-sites present in all samples, excluding MSD-sites with missing data, circumventing the variation in DNA quality between samples.

Using MS-DArT-seq, methylation is called based on the difference of library specific (*Msp*I and *Hpa*II) counts of mapped reads against a reference genome. It is important to highlight that the availability of a reference genome is optional, making the methodology applicable to species poor in genomic information. Furthermore, the technique showed high reproducibility, as demonstrated by the strong correlation for the three biological replicates of each sample tissue analyzed, both in terms of site sampling and methylation calling (S2 Fig).

MS-DArT-seq was confronted with the current gold standard protocol for cytosine methylation detection at single nucleotide resolution, WGBS. Taking the adult leaves sample as validation data, the majority of the MSD-methylated sites detected by our approach was confirmed by WGBS results (95.47%; S1 File), endorsing the great accuracy in DNA methylation detection promoted by the combination of the experimental method MS-DArT-seq data and our analytical pipeline.

Regarding the functional annotation of MSD-tags, we observe a sampling bias towards genomic sites positioned inside annotated genes. Strikingly, around 47% of our MSD-sites (15,317 out of 32,357) are located in genes, albeit the bulk majority of repeats and non-genic regions (Table 1; Figs 5 and 6). This can be a result of the chosen REs. Even though *Msp*I is considered to be methylation insensitive, it was shown that both *Msp*I and *Hpa*II are blocked by methylation in the external cytosine (C^ext^) of the restriction site 5’-C^ext^C^int^GG-3’, which corresponds to the CHG context observed in plants [39,41]. Therefore, methylated CHG sites are not sampled by MS-DArT-seq and, since CHG methylation occurs more frequently in intergenic regions and is predominantly enriched in repetitive elements [43], these locations tend to be filtered out favoring genic region sampling. The enzyme *Pst*I also shows sensitivity to CHG methylation, enhancing this effect [41,44]. A potential disadvantage of this *Pst*I attribute is the reduction in the number of sampled sites. As shown in Fig 3A), while thousands of MSD-sites were identified, a considerable fraction of the fragments predicted *in silico* were not sequenced, potentially due to avoidance of hypermethylated regions and DNA methylation in the CHC context. Moreover, the removal of multiple mapped reads can promote the underrepresentation of sequenced fragments originated from genomic regions rich in repetitive elements, contributing to the reduction in the number of sampled sites by our analytical pipeline.

The genic region bias of MS-DArT-seq may be favorable to appraise the effects of methylation dynamics in gene expression, imprinting and gene body methylation, although not in a targeted way. For instance, a survey based on WGBS determined that ~21% of *E*. *grandis* genes (7,731 of 36,349) showed methylation patterns indicative of GBM [43]. In our experimental setting, 45.48% of all genes with GBM in *E*. *grandis* were sampled by MS-DArT-seq, suggesting that this technique provides an attractive alternative to study this puzzling biological phenomenon [12], at least in plants, at a fraction of the cost of WGBS.

In terms of methylated TEs, MS-DArT-seq is able to identify a fraction of the elements in each of the main classes (Fig 7), although there is a greater than expected chance to detect subsets of TEs annotated as HELITRON, LINE and Nested TEs, whereas LARD and MITE elements were less frequently detected as methylated (S8 Fig). This observation is consistent in all tissues, but neither the reasons for this discrepancy nor its generalization to other species can be ascertained at this stage.

Another interesting observation was the significant difference observed in methylation patterns between immature leaves and the two other samples coming from mature tissues (leaves and xylem), that may be explained by the dynamic feature of DNA methylation during developmental stages [45–49]. Either increase [50,51] or decrease [52] in methylation levels has been observed in different species as tissues mature. Other evidence come from elevated expression of the RdDM (RNA directed DNA methylation) pathway and other chromatin regulators in meristems suggesting they act as a relay mechanism to ensure correct propagation of silent states to new tissues and organs [53], from epigenomic diversity observed between cell types within root meristem [54] and from highly dynamic DNA methylation during cork cells differentiation [55]).

To the best of our knowledge, there are only two other published computational methods for DNA methylation data analysis based on methyl-sensitive restriction enzymes combined with NGS. Chwialkowska et al. [40] developed MSAP-Seq and Mayne et al. [56] developed the R package msgbsR, which employs a similar approach. However, these methods are constrained to MSCC-type data, dealing only with frequent cutter enzymes, which is not applicable to the double digestion strategy adopted by MS-DArT-seq. Overall, our pipeline provides a broad and reliable overview of DNA methylation profile on *Msp*I/*Hpa*II restriction sites and can handle the double digestion scenario. Also, the aforementioned programs can only determine a sample methylation status contrasting to another sample (usually, a control condition). Since our approach is based on direct comparison between *Pst*I-*Msp*I and *Pst*I-*Hpa*II libraries for each sample, it is possible to determine the methylation status in thousands of genomic sites independently of a control sample.

In the light of probing the dynamic context of epigenetic states in cells, the profusion of DNA methylation profiling methods arises from the necessity to equate resolution, scalability and costs. Whole genome bisulfite-based approaches provide the best coverage and a quantitative view of methylated cytosines in a genome. But this comes at a high price tag and with the strict requirement of a reference genome, that hinders its application for the vast majority of species. Restriction-based methods, despite the noticeable sampling distortion, offer an affordable gateway to epigenomic studies in species with poor genomic resources. MS-DArT-seq is a new representative of this approach, allowing DNA methylation detection with accuracy and scale. We suggest this approach can be of great importance in population studies, balancing large sample sizes with a discrete, but informative, overview of the epigenome.

## 4. Materials and methods

### 4.1 Plant material

Plant material used in this study are three *Eucalyptus grandis* trees which are biological replicates or clones of the genotype BRASUZ1, the same used for the sequencing of the species reference genome [35]. The clones are planted at EMBRAPA (Brazilian Agricultural Research Corporation) Genetic Resources and Biotechnology, Brasília, Brazil. Three tissues were collected simultaneously from the trees of 4 year old: completely developed leaves referred to as adult leaves, juvenile or developing leaves (for each tree, both leaves tissues were sampled from the same branch which was in the side of the tree directed to sunlight exposition) and xylem. Genomic DNA of each sample was extracted using an adapted protocol based on the classic CTAB [57,58] method. MS-DArT library preparation and sequencing was carried by the Diversity Arrays Technology Ltd. (DArT, Australia).

### 4.2 Methylation sensitive DArT sequencing (MS-DArT-seq)

DArT-seq represents a combination of a DArT complexity reduction method and next generation sequencing platforms [32]. For methylation analysis, named MS-DArT-seq, two parallel libraries are prepared per sample using DNA double digestion restriction-based protocol with the combination of the enzymes *Pst*I and *Msp*I (*Pst*I-*Msp*I) and *Pst*I and *Hpa*II (*Pst*I-*Hpa*II). DNA samples are processed in digestion/ligation reactions as described in [32], except for replacing a single *Pst*I-compatible adaptor with two different adaptors corresponding to two different Restriction Enzyme (RE) overhangs. Therefore, only “mixed fragments” (*Pst*I-*Msp*I and *Pst*I-*Hpa*II) are effectively amplified in 30 rounds of PCR. Then, the amplification products are single-end sequenced in an Illumina Hiseq 2500 machine for 77 cycles.

### 4.3 Data processing and sampled sites definition

Quality check of MS-DArT-seq raw sequence data was performed by FastQC (http://www.bioinformatics.babraham.ac.uk/projects/fastqc/). Low quality bases and sequencing adapters were trimmed using Trimmomatic v.0.36 [59] with default parameters, except for the addition of *SLIDINGWINDOW*:*5*:*25* option. Read mapping was performed by bowtie2 v.2.3.5 [60]. Reads from all libraries were mapped together on the *Eucalyptus grandis* genome v.2.0 (Phytozome v.12; Goodstein et al. [61]) in four steps. At first, no mismatches were allowed and at each subsequent step only unmapped reads were used, allowing one additional mismatch at a time up to three mismatches. No gaps were allowed and reads mapped to multiple locations were removed.

Bedtools v.2.27.1 [62] was used to extract the positions of mapped reads. This information was processed by an in-house R script to determine the sequenced fragments, named as MSD-tags, as well as, the position of the corresponding *Msp*I/*Hpa*II restriction sites, named MSD-sites, whose methylation status are interrogated by reads counts comparison between the two libraries. Both MSD-tags and MSD-sites are determined using the mapped reads locations and positions of the *Msp*I/*Hpa*II and *Pst*I restriction sites in the *E*. *grandis* reference genome, as described in Fig 1.

The distribution of sequenced fragments (MSD-tags) was compared with the distribution of *in silico* predicted fragments by the genomic digestion using the combination of *Msp*I/*Hpa*II and *Pst*I restriction enzymes. To test if these two distributions are comparable, we used a two-sample Kolmogorov-Smirnov test. Since these are discrete distributions, the bootstrap Kolmogorov-Smirnov test (Matching R package [63]) was applied using one thousand Monte Carlo simulations to determine the proper p-value.

The program Ologram [36] was applied to evaluate the statistical significance of colocalization between the *in silico* fragments generated by the double digestion using *Pst*I-*Msp*I/*Hpa*II and genes. Similarly, a colocalization analysis was also conducted for *in silico* fragments generated by double digestion using the restriction enzymes *Bam*HI and *Eco*RI and *Msp*I/*Hpa*II as alternatives to *Pst*I. For all colocalization analysis, the *in silico* digestion was performed by a custom R script. Fragments smaller than 100 bp or larger than 600 bp were removed since they are outside the fragment size range for Illumina sequencing library construction.

To test if the distribution of the MSD-sites along the chromosomes of *E*. *grandis* is uniform, each chromosome was split in windows of different sizes (1 Mb, 500 Kb, 250 Kb and 100 Kb) and a *goodness-of-fit* test (chi-squared) was applied using the number of sampled sites in each window.

To identify the methylated sites (MSD-methylated sites) in each sample, libraries (*Pst*I-*Msp*I and *Pst*I-*Hpa*II) reads were mapped individually to the *E*. *grandis* assembly and counts for each MSD-tag were found by featureCounts v.1.6.2 [64].

### 4.4 DNA methylation determination by reads count comparison

For each sample in this study, determination of MSD-methylated sites was carried out by comparison between counts of each MSD-tag in *Pst*I-*Hpa*II and *Pst*I-*Msp*I libraries. Bioconductor packages edgeR [37] and DEseq2 [38] were used for this comparative analysis, assuming that counts follow a negative binomial distribution. Adult leaves MS-DArT-seq data was used as a reference to determine the optimized values of the False Discovery Rate (FDR) limit, minimal Fold Change between counts from *Pst*I-*Msp*I and *Pst*I-*Hpa*II libraries and minimal counts necessary for a MSD-tag to be considered not spurious (minimal count filter), since bisulfite sequencing was accomplished for the same sample (see below). These three parameters were used to filter significant differences between counts of *Pst*I-*Hpa*II and *Pst*I-*Msp*I libraries.

In a population of different cells types, the same restriction site can be found in distinct methylation states. Therefore, different fragments can be produced from the same correspondent region in different cells, depending on the site accessibility to the RE. As a consequence, where multiple *Msp*I/*Hpa*II sites are close to each other in the genome, their correspondent MSD-tags can overlap (S9 Fig). We try to account for that during the MSD-tag definition and counts of each MSD-tag were adjusted (S9 Fig). Also, we consider reads always starting in a *Pst*I site.

Three values of the FDR (≤ 0.05, ≤ 0.01 and ≤ 0.001), two values of the Fold Change (≥ 1 and ≥ 2) and three values of the minimal counts filter (≥ 3, ≥ 5 and ≥ 10) were tested and all combinations among those parameters were evaluated. For each combination, identified MSD-methylated sites were compared with the WGBS data correspondent cytosines (S1 File). The combination of these parameters with the largest number of MSD-methylated sites in the validation sample (adult leaves), alongside with a maximized WGBS validation rate (called as methylated when truly methylated), was used to determine MSD-methylated sites of all MS-DArT-seq samples on this study.

### 4.5 DNA Methylation patterns of BRASUZ1 tissues

To compare methylation patterns of three different tissue samples of BRASUZ1 tree, it was necessary to ensure that only MSD-sites sampled in all tissues would be employed. Only MSD-tags with one or more reads in *Pst*I-*Msp*I library of all tissues were submitted to edgeR and DEseq2 algorithms. The genomic context of MSD-methylated sites for each tissue was evaluated to determine if methylations were positioned within or close to genes and/or on transposable elements (TEs). Both TEs and genes which contain at least one of the MSD-methylated sites, referred as methylated TEs and methylated genes, were annotated and comparisons of these elements among tissues were performed.

Cochran’s Q test was applied to test if the probability of occurrence of a methylated site differs among tissues. If the p-value was significant, pairwise comparisons were carried out using the Wilcoxon sign test. The same approach was used to determine if the probability of each gene and TE have at least one MSD-methylated site is equal among the BRASUZ1 tissues sampled.

### 4.6 Gene functional annotation and TEs classification

Functional annotation of all methylated genes was executed by the softwares Blast2GO v,4.1 [65] and BioMart [66] (biomaRt R package [67]), using Mart provided by Phytozome v.12. For each gene, annotation provided by the two databases were merged. To verify if there is over-representation of methylated genes related to specific biological functions or aspects, enrichment analysis of Gene Ontology (GO) terms was performed using the Bioconductor clusterProfiler package [68]. Prediction and classification of transposable elements was performed by REPET v2.5 package [69].

### 4.7 Bisulfite sequencing

#### 4.7.1 Library construction

Libraries were prepared by Zymo Research using Methyl-MaxiSeq protocol. Starting with the digestion of 500 ng of genomic DNA with 2 units of Zymo Research’s (ZR) dsDNA Shearase TM Plus (Cat#: E2018-50), fragments were end-blunted and 3’-terminal-A extended, and then purified using the Zymo Research (ZR) DNA Clean & Concentrator TM– 5 kit (Cat#: D4003). The A-tailed fragments were ligated to pre-annealed adapters containing 5’-methyl-cytosine instead of cytosine and adapter-ligated fragments were filled-in. Bisulfite treatment of the fragments was done using the EZ DNA Methylation–Lightning kit (ZR, Cat#: D5030). PCR was performed with Illumina TruSeq indices, size and concentration of fragments were confirmed on the Agilent 2200 TapeStation and then sequenced on Illumina Hiseq.

#### 4.7.2 Sequence alignments and data analysis

Sequence reads from bisulfite-treated EpiQuest libraries were identified using standard Illumina base-calling software and analyzed with a Zymo Research proprietary analysis pipeline written in Python and using Bismark [70] as the alignment software for analysis. Index files were constructed by bismark_genome_preparation command using the *E*. *grandis* reference genome. Default parameters and—non_directional were applied while running Bismark. Methylation level of each sampled cytosine was estimated as the number of reads reporting a C, divided by the total number of reads reporting a C or T.

To call methylated sites, we applied an approach similar to the one described by Schultz et al. [71]. A binomial test was applied using the number of reads that supported methylation at a site as the number of success and total reads at the same site as the number of trials. The probability of success was given by the WGBS non-conversion rate and p-values were corrected by the False Discovery Rate (FDR) procedure (Benjamini–Hochberg). A cytosine was considered methylated if the associated FDR was ≤ 0.01 and there were at least three reads supporting this position.

To validate MSD-methylated sites (verified in the adult leaves sample) using WGBS data, only MSD-sites (5’-CCGG-3’) where both internal cytosines, in the Watson and Crick strands, were supported for at least 3 reads in the WGBS were considered. Using this comparison, it was possible to classify MSD-methylated sites as fully-methylated (both the internal cytosines methylated) or hemi-methylated (one of the internal cytosines methylated).

### 4.8 Availability of data and materials

Raw data produced by MS-DArT-seq sequencing was deposited in figshare database and is available to download at https://doi.org/10.6084/m9.figshare.10305431. Bioinformatic analyses were performed by open-source software and by Python and R scripts elaborated in the context of this study. The analytical steps are coordinated and executed by the Snakemake workflow management system [72]. Scripts and the snakemake configuration files are available in GitHub at https://github.com/wendelljpereira/ms-dart-seq.

### 4.9 Statistical analysis

Besides the software described in the previous sections to process the sequencing data, all analysis, statistical tests and plots generation were conducted using R programming language v.3.5.1. A list of all R packages, their respective versions, as well as the source code is available at https://github.com/wendelljpereira/ms-dart-seq.

Cochran’s Q non-parametric test was applied to compare the methylation state of MS-Tags (yes or no) matched across the tissues, with the null hypothesis considering that the proportion of methylated sites is the same for all tissues. In case of rejection of Cochran’s Q test null hypothesis, group differences were tested by pairwise comparisons using Wilcoxon sign test.

Fisher’s exact test (one-sided) was applied to determine if MS-DArT-seq sampling is biased toward genic space, generating more MSD-sites in genic regions than expected. Also, the same test was applied to determine if the MSD-Methylated sites are more frequent in MSD-sites of genes than in MSD-sites located outside genes.

For all statistical tests we considered 5% as the significance threshold and the false discovery rate (FDR) procedure (Benjamini–Hochberg) was used to correct for multiple comparisons.

## Supporting information

S1 Fig Distribution of the 32,357 MSD-sites, with counts in all samples, along the *E*. *grandis* chromosomes. Each bar represents a window of 250 kilobases.
(TIFF)

S2 Fig Correlation plot of biological replicates for each tissue. a) Pearson correlation coefficients of the comparisons between replicates counts in *Pst*I-*Msp*I libraries. b) Pearson correlation coefficients of the comparisons between replicates counts in the *Pst*I-*Hpa*II libraries. The order of samples was determined by hierarchical clustering.
(TIFF)

S3 Fig Principal components analysis (PCA) of the MSD-tags considered as MSD-methylated sites (which counts differ significantly between *Pst*I-*Msp*I and *Pst*I-*Hpa*II libraries). (TIFF)

S4 Fig Association plot demonstrating Pearson’s residuals of the independence model (chi-squared) applied to the distribution of MSD-methylated sites in the vicinity of genes (10 kb) compared with the distribution of *Msp*I sites in the same region. (TIFF)

S5 Fig Venn diagrams demonstrating comparison among the MSD-methylated sites of three tissues. In a) and b) are presented the comparisons of MSD-methylated sites in genes and in TEs, respectively. Comparison of methylated genes and TEs, here defined as genes or TEs that contains at least one of the MSD-methylated sites, are respectively demonstrated in c) and d) plots. All comparisons are supported by Cochran’s Q and Wilcoxon sign tests which demonstrated that each tissue is significantly different from the other two (p-value < 0.05).
(TIFF)

S6 Fig Association plot demonstrating residuals (Pearson) of the independence model (chi-squared) applied to the distribution of MSD-methylated sites per genomic features for each tissue. The shown p-value in the bottom right was generated by Pearson’s chi-squared test of a multi-way contingency table and rejects (p<0.05) the null hypothesis of complete independence among the samples. The area of each box is proportional to the difference in observed and expected frequencies and the color shading highlights the individual cells that are probably individually significant. While the color shading is helpful as a proxy of significance it should be interpreted as an indicator of higher deviation from the expected.
(TIFF)

S7 Fig Classification of all TEs in *E*. *grandis* genome (left) and methylated TEs in each sample (right). (TIFF)

S8 Fig Association plot demonstrating residuals (Pearson) of the independence model (chi-squared) applied to the distribution of methylated TEs in classes for each sample alongside the distribution of all TEs in *E*. *grandis* genome. (TIFF)

S9 Fig Counts correction of regions where there are overlaps of MSD-tags. Because the same restriction site can be blocked (methylated in the external cytosine) or accessible in different cells that are part of the pool sampled, different patterns of DNA fragments are produced. Likewise, multiple MSD-tags could be generated in the same region, each one related to a different restriction site (MSD-site). Due to these overlaps, the count of the larger MSD-tag is also attributed to the ones inserted in the same region. After the correction, the counts of each tag should match their correspondent MSD-site, improving the detection of DNA methylation.
(TIFF)

S10 Fig Colocalization analysis of *in silico* fragments with gene features (CDS, exon and UTR) and intergenic regions. Statistics of colocalization of *in silico* fragments generated by digestion with different combinations of REs and genomic features (genic and intergenic regions), as evaluated by the software Ologram [36]. In yellow, it is shown the observed intersections for the set of fragment intervals; in blue, intersections of the random shuffled regions. Error bars represent the standard deviation of the shuffled distribution. The p-values for each feature colocalization is shown above the category bars. A-D) Statistics of colocalization as calculated by the number of bases in the intersection between regions. E-F) Statistics of colocalization as calculated by the number of intersections between regions.
(TIFF)

S1 File Descriptive statistics of the comparison between MS-DArT-seq and WGBS using different parameters for MS-DArT-seq methylation calling. (XLSX)

S2 File Functional annotation of genes containing MSD-methylated sites organized by tissue. (XLSX)

S3 File Enriched GO terms of genes containing MSD-methylated sites organized by tissue. (XLSX)

S1 Table Contingency tables used in Fisher’s exact test to determine if MS-DArT-seq sampling is biased toward genes (A) and to determine if detection of methylation is independent of the genomic context (B). In A) is represented the distribution of all 979,886 *Msp*I/*Hpa*II restriction sites in the genome (CCGG). A fraction of those was sampled by MSD-DArT-seq and called MSD-sites. The contingency table shows the proportion of all sites located in genes and in intergenic regions, expliciting the ones sampled (MSD-sites) or not sampled by MSD-DArT-seq in our study. The applied Fisher’s exact test evaluates if sites both in genic and intergenic regions have an equal chance of being sampled by this technology (the null hypothesis that sampling is independent of the genomic location). Therefore, since the null hypothesis was rejected (p-value < 0.001), against an alternative hypothesis of true odds ratio greater than 1, it is possible to demonstrate that *Msp*I/*Hpa*II restriction sites in genes are more likely to be selected by MSD-DArT-seq than in intergenic regions. In B), it is shown the distribution of the MSD-sites with no missing data in all tissues, after correction of tags redundancy (31,427 MSD-sites). The contingency table shows the proportion of these sites, located in genes and intergenic regions, grouped by their methylation status. Similarly to A, the rejection of the null hypothesis indicates that MSD-sites in genes have a higher probability of being methylated than MSD-sites in intergenic regions, even though MSD-sites in intergenic are the most abundant.
(DOCX)
