# Supplementary figures and images for: A cost-effective approach to DNA methylation detection by Methyl Sensitive DArT sequencing
Source: PLoS One. 2020 Jun 4;15(6):e0233800. doi: 10.1371/journal.pone.0233800 (PMC7272069; doi:10.1371/journal.pone.0233800)

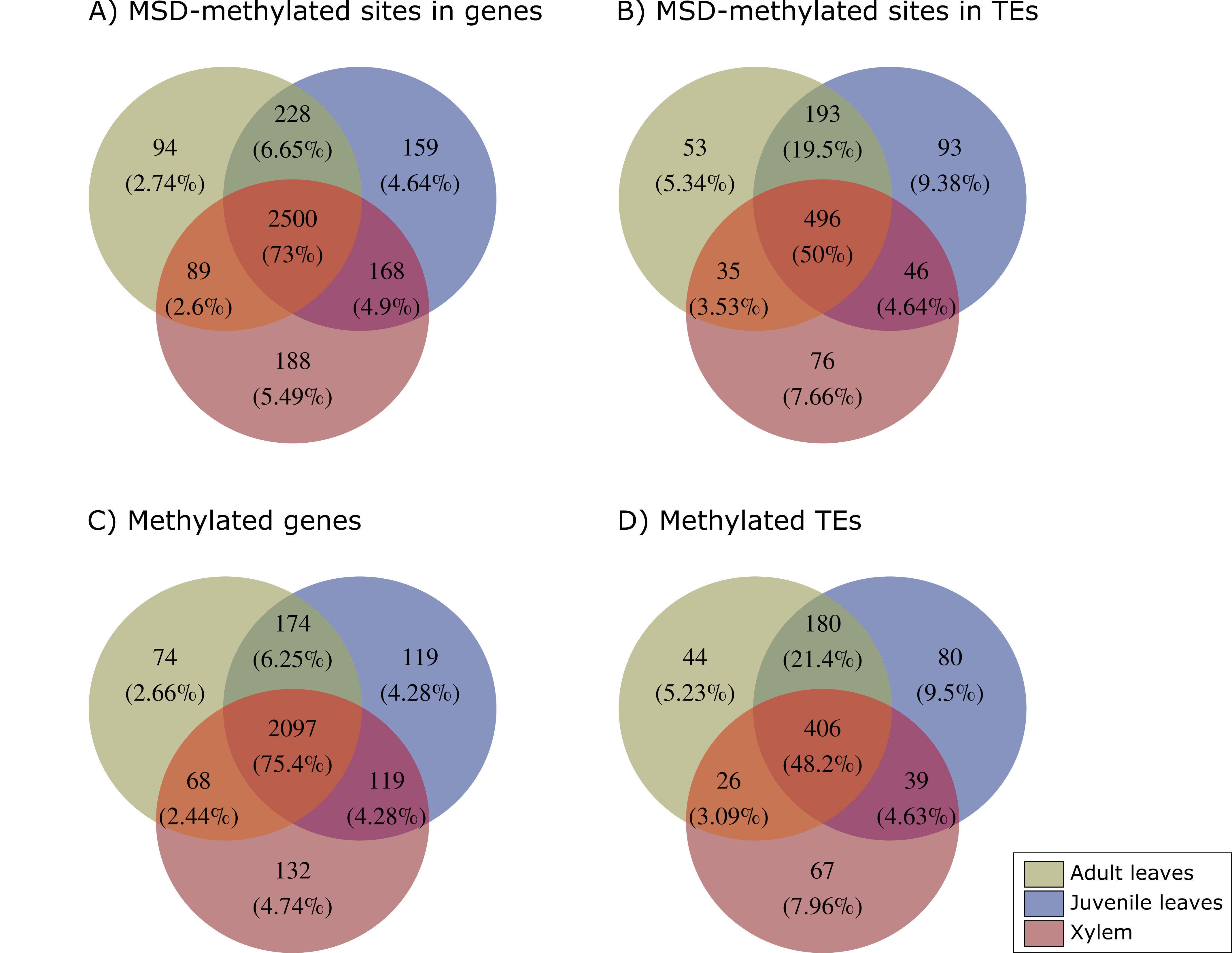

Supplement: S5 Fig — In a) and b) are presented the comparisons of MSD-methylated sites in genes and in TEs, respectively. Comparison of methylated genes and TEs, here defined as genes or TEs that contains at least one of the MSD-methylated sites, are respectively demonstrated in c) and d) plots. All comparisons are supported by Cochran’s Q and Wilcoxon sign tests which demonstrated that each tissue is significantly different from the other two (p-value < 0.05). (TIFF) [file pone.0233800.s005.tiff]

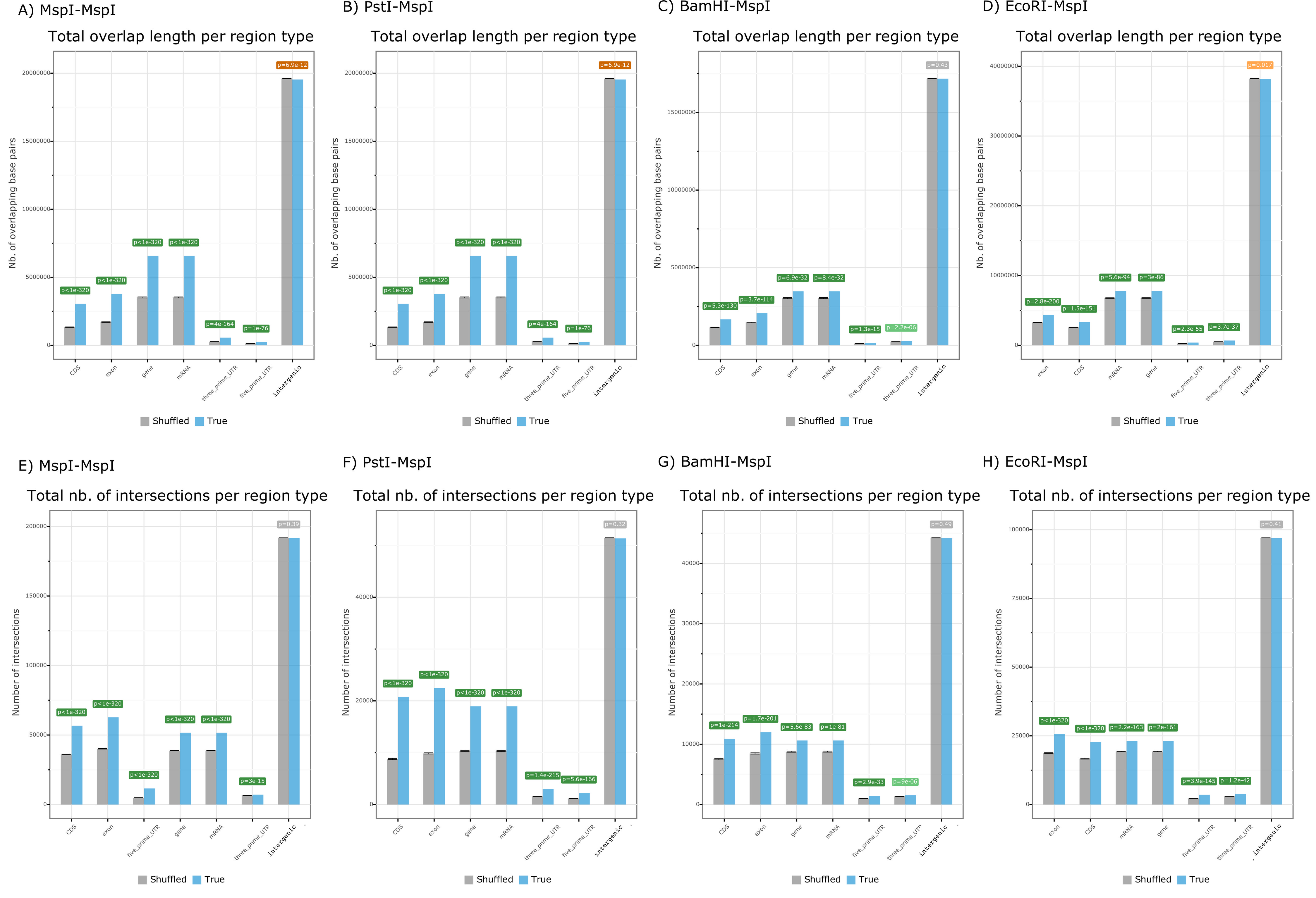

Supplement: S10 Fig — Statistics of colocalization of in silico fragments generated by digestion with different combinations of REs and genomic features (genic and intergenic regions), as evaluated by the software Ologram [36]. In yellow, it is shown the observed intersections for the set of fragment intervals; in blue, intersections of the random shuffled regions. Error bars represent the standard deviation of the shuffled distribution. The p-values for each feature colocalization is shown above the category bars. A-D) Statistics of colocalization as calculated by the number of bases in the intersection between regions. E-F) Statistics of colocalization as calculated by the number of intersections between regions. (TIFF) [file pone.0233800.s010.tiff]
