# Supplementary material for: A cost-effective approach to DNA methylation detection by Methyl Sensitive DArT sequencing
Source: PLoS One. 2020 Jun 4;15(6):e0233800. doi: 10.1371/journal.pone.0233800 (PMC7272069; doi:10.1371/journal.pone.0233800)
Supplement: S1 Table — In A) is represented the distribution of all 979,886 MspI/HpaII restriction sites in the genome (CCGG). A fraction of those was sampled by MSD-DArT-seq and called MSD-sites. The contingency table shows the proportion of all sites located in genes and in intergenic regions, expliciting the ones sampled (MSD-sites) or not sampled by MSD-DArT-seq in our study. The applied Fisher’s exact test evaluates if sites both in genic and intergenic regions have an equal chance of being sampled by this technology (the null hypothesis that sampling is independent of the genomic location). Therefore, since the null hypothesis was rejected (p-value < 0.001), against an alternative hypothesis of true odds ratio greater than 1, it is possible to demonstrate that MspI/HpaII restriction sites in genes are more likely to be selected by MSD-DArT-seq than in intergenic regions. In B), it is shown the distribution of the MSD-sites with no missing data in all tissues, after correction of tags redundancy (31,427 MSD-sites). The contingency table shows the proportion of these sites, located in genes and intergenic regions, grouped by their methylation status. Similarly to A, the rejection of the null hypothesis indicates that MSD-sites in genes have a higher probability of being methylated than MSD-sites in intergenic regions, even though MSD-sites in intergenic are the most abundant. (DOCX) [file pone.0233800.s014.docx]

**Table S1**. **Contingency tables used in Fisher’s exact test to determine if MS-DArT-seq sampling is biased toward genes (A) and to determine if detection of methylation is independent of the genomic context (B).** In A) is represented the distribution of all 979,886 *Msp*I/*Hpa*II restriction sites in the genome (CCGG). A fraction of those was sampled by MSD-DArT-seq and called MSD-sites. The contingency table shows the proportion of all sites located in genes and in intergenic regions, expliciting the ones sampled (MSD-sites) or not sampled by MSD-DArT-seq in our study. The applied Fisher’s exact test evaluates if sites both in genic and intergenic regions have an equal chance of being sampled by this technology (the null hypothesis that sampling is independent of the genomic location). Therefore, since the null hypothesis was rejected (p-value < 0.001), against an alternative hypothesis of true odds ratio greater than 1, it is possible to demonstrate that *Msp*I/*Hpa*II restriction sites in genes are more likely to be selected by MSD-DArT-seq than in intergenic regions. In B), it is shown the distribution of the MSD-sites with no missing data in all tissues, after correction of tags redundancy (31,427 MSD-sites). The contingency table shows the proportion of these sites, located in genes and intergenic regions, grouped by their methylation status. Similarly to A, the rejection of the null hypothesis indicates that MSD-sites in genes have a higher probability of being methylated than MSD-sites in intergenic regions, even though MSD-sites in intergenic are the most abundant.

| **A) MS-DArT-seq sampling** | | |
| --- | --- | --- |
|  | **Sampled *Msp*I/*Hpa*II sites (MSD-sites)** | **Not sampled *Msp*I/*Hpa*II sites** |
| **Gene** | 29,552 | 165,890 |
| **Intergenic** | 42,963 | 741,481 |
| **B) Detection of DNA methylation from MSD-sites common to all tissues** | | |
|  | **MSD-methylated sites** | **Unmethylated MSD-sites** |
| **Gene** | 3,532* | 11,478 |
| **Intergenic** | 2,007 | 14,103 |

* MSD-methylated sites in TEs inside an intron or UTR of a gene were considered as MSD-methylated sites in genes. Also, MSD-methylated sites classified as “features overlaps” (307) were not used in this test.
